# Mitochondrial disease in adults: what's old and what's new?

**DOI:** 10.15252/emmm.201505079

**Published:** 2015-11-26

**Authors:** Patrick F Chinnery

**Affiliations:** ^1^ Department of Clinical NeurosciencesSchool of Clinical MedicineUniversity of CambridgeCambridgeUK; ^2^ Medical Research Council – Mitochondrial Biology UnitCambridge Biomedical CampusCambridgeUK

**Keywords:** mitochondrial disease, mitochondrial DNA, mitochondrial encephalomyopathy, myopathy, neurometabolic, Genetics, Gene Therapy & Genetic Disease, Metabolism

## Abstract

Ten years ago, there was an emerging view that the molecular basis for adult mitochondrial disorders was largely known and that the clinical phenotypes had been well described. Nothing could have been further from the truth. The establishment of large cohorts of patients has revealed new aspects of the clinical presentation that were not previously appreciated. Over time, this approach is starting to provide an accurate understanding of the natural history of mitochondrial disease in adults. Advances in molecular diagnostics, underpinned by next generation sequencing technology, have identified novel molecular mechanisms. Recently described mitochondrial disease phenotypes have disparate causes, and yet share common mechanistic themes. In particular, disorders of mtDNA maintenance have emerged as a major cause of mitochondrial disease in adults. Progressive mtDNA depletion and the accumulation of mtDNA mutations explain some of the clinical features, but the genetic and cellular processes responsible for the mtDNA abnormalities are not entirely clear in each instance. Unfortunately, apart from a few specific examples, treatments for adult mitochondrial disease have not been forthcoming. However, the establishment of international consortia, and the first multinational randomised controlled trial, have paved the way for major progress in the near future, underpinned by growing interest from the pharmaceutical industry. Adult mitochondrial medicine is, therefore, in its infancy, and the challenge is to harness the new understanding of its molecular and cellular basis to develop treatments of real benefit to patients.

**Glossary**
31P‐MRS: Phosphorus magnetic resonance spectroscopy is a functional MRI technique that can be used to indirectly measure mitochondrial ATP synthesis in skeletal and cardiac muscle *in vivo*
Heteroplasmy (and the threshold effect): A mixture of wild‐type and mutated mtDNA. At a cellular level, the percentage level of heteroplasmy determines whether there is a biochemical defect affecting oxidative phosphorylation and ATP synthesis
Homoplasmy: When all mtDNA molecules are identical
Kearns‐Sayre syndrome: A clinical syndrome involving progressive external ophthalmoplegia, ptosis (drooping eyelids), pigmentary retinopathy, cardiac conduction abnormalities, ataxia, an elevated cerebrospinal fluid protein, diabetes mellitus, sensorineural hearing loss and myopathy
LHON: Leber hereditary optic neuropathy. A mtDNA disorder causing maternally inherited blindness that typically develops in mid‐adult life and preferentially affects men
MELAS: Mitochondrial myopathy, encephalopathy, lactic acidosis and stroke‐like episodes. A classical mitochondrial disorder usually due to the m.3243A>G mtDNA mutation
MNGIE: Mitochondrial neurogastrointestinal encephalopathy. A rare autosomal recessive disorder caused by mutations in *TP,* which codes for thymidine phosphorylase
MRI: Magnetic resonance imaging
mtDNA depletion disorders: Autosomal recessive diseases causing a reduction in the amount of mtDNA
mtDNA maintenance disorders: An emerging and complex group of nuclear genetic mitochondrial disorders which cause secondary point mutations or deletions of mtDNA. The secondary mtDNA abnormalities contribute to the clinical features of the disease
mtDNA: Human mitochondrial DNA is a 16.5‐Kb molecule of double‐stranded DNA. MtDNA codes for 13 respiratory chain proteins and both tRNA and RNAs required by the mitochondrion for intra‐mitochondrial protein synthesis. Multiple copies of mtDNA are present in each cell. The number is tightly regulated and cell specific
Oxidative phosphorylation: Metabolic pathway on the inner mitochondrial membrane which generates ATP through the oxidation of cofactors generated by intermediary metabolism
PEO: Progressive external ophthalmoplegia. A clinical syndrome with weakness of the external eye muscles, often accompanied by ptosis (drooping eyelids)
POLG: Nuclear gene encoding the mitochondrial DNA polymerase γ

## Introduction

**Figure 1:**
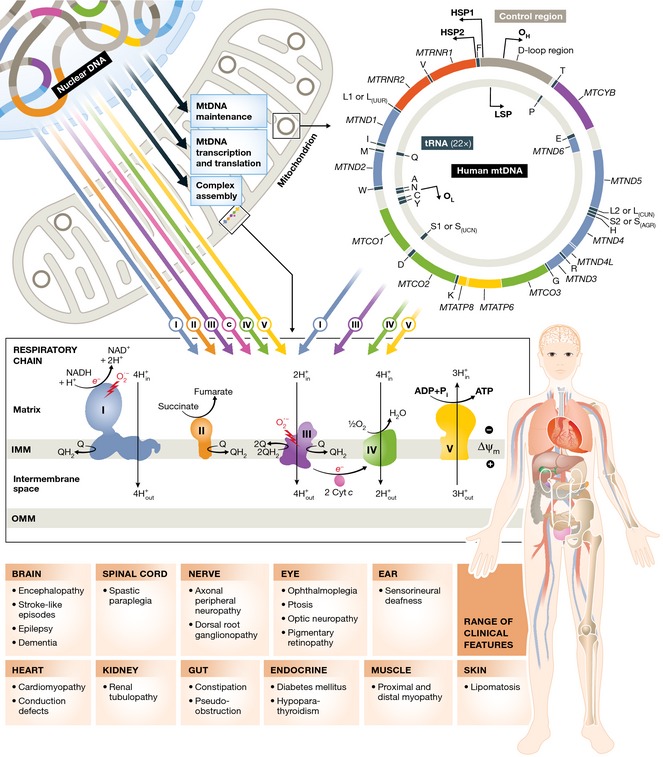
Mitochondrial biogenesis and the clinical features of mitochondrial disease in adults Upper panel: Adenosine triphosphate (ATP) is generated by the process of oxidative phosphorylation. This is achieved by the concerted action of ~90 proteins arranged into five respiratory chain complexes on the inner mitochondrial membrane. Thirteen of these proteins are encoded by the mitochondrial genome (mtDNA, right), which is present in high copy number in the mitochondrial matrix (100s to 1,000s per cell, depending on the cell type). The remaining mitochondrial proteins are synthesised in the cytoplasm from nuclear gene transcripts (left) and include the remaining structural subunits; complex assembly factors; proteins involved in the replication, maintenance and expression of mtDNA; and functional and structural components of the mitochondrial membrane. Mutations in genes encoding all of these proteins can cause mitochondrial diseases. Lower panel: the range of clinical features varies from patient to patient. Some have only one or a few of the features listed, whereas other patients have many in a multi‐system disease. Although some genetic defects cause specific phenotypes (e.g. the mtDNA mutations causing Leber hereditary optic neuropathy, which principally affect a single cell type in the vast majority of patients), other genetic defects cause an overlapping spectrum of phenotypes that can be caused by mtDNA and nuclear DNA mutations. The reasons for the tissue selectivity are not well understood.

The year 1988 saw the first description of patients with pathogenic mutations of mitochondrial DNA (mtDNA) (Holt *et al*, 1988; Wallace *et al*, 1988), opening the floodgates, and paving the way for the new discipline of clinical mitochondrial medicine. The following decades saw major advances in our understanding of the molecular basis of mitochondrial disorders, initially focused on the smaller, more tractable mitochondrial genome. In later years, this was accompanied by the identification of novel nuclear gene defects (Koopman *et al*, 2012), which have emerged as a major cause of mitochondrial disorders (Gorman *et al*, 2015c). Review articles published a over decade ago described a molecular dichotomy between adults and children (Leonard & Schapira, 2000a,b), with mtDNA defects typically presenting in adult life, and children typically having autosomal recessive disorders due to presumed defects of the nuclear DNA. Over the last 10 years, it has become clear that mtDNA mutations can present throughout life and that autosomal recessive, dominant and X‐linked nuclear genetic disorders frequently present in adult life. This is but one of many examples where our initial impression of a clinical genotype–phenotype relationship has become blurred as our knowledge base has increased. Broad, overlapping phenotypes are caused by a myriad of molecular lesions affecting both genomes that present throughout the human life course (Fig 1). One could argue that separate review articles on childhood and adult mitochondrial diseases simply perpetuate the old dogma. However, despite this complexity, certain patterns do still exist, linking curious facets of the clinical presentation with associated biochemical abnormalities and their underlying molecular defects. All of this is, however, work in progress—and it remains to be seen whether the strict separation of childhood and paediatric mitochondrial disease will stand the test of time.

This review focuses specifically on recent insight gained in mitochondrial disorders presenting predominantly in adult life. The huge growth of literature means that it cannot be comprehensive, but the examples illustrate some emerging principles.

## Epidemiology

Until 1999, it was generally thought that mitochondrial disorders were extremely rare, perhaps affecting a few patients in every million of the population. However, a number of epidemiological studies performed in Europe and the antipodes challenged this view (Majamaa *et al*, 1998; Chinnery *et al*, 2000; Skladal *et al*, 2000; Darin *et al*, 2001). Initially focusing on the presence of mtDNA‐related disease, the most up to date figures for 2015 incorporate both mitochondrial DNA and nuclear DNA mutations, which are found in approximately 1:4,300 of the population (Gorman *et al*, 2015c). In the north of England, 23% of adults with mitochondrial disease had known or presumed nuclear genetic causes for their mitochondrial disease, with the most prevalent being mutations in *SPG7* (Pfeffer *et al*, 2015), closely followed by *PEO1* and *OPA1* (Yu‐Wai‐Man *et al*, 2010a; Gorman *et al*, 2015c). For mtDNA, point mutations causing Leber's hereditary optic neuropathy (LHON, m.11778G>A, m.3460G>A and m.14484T>C) were found in ~40% of patients affected by mitochondrial disease (Man *et al*, 2003; Gorman *et al*, 2015c). Overall, these recent findings support the original view that mtDNA mutations are the major cause of mitochondrial disease in adults, but demonstrate clearly the importance of a diverse array of nuclear gene defects that can present to the clinic in adult life. The implication of these findings is profound. Different mutations are associated with different disease features and progress at different rates, and defining the genetic basis of the mitochondrial disorder has important implications for genetic counselling and prenatal diagnosis.

## New phenotypes–new genotypes

### Refined phenotypes

With the widespread availability of molecular diagnostic tests, increasing numbers of patients have been diagnosed worldwide. A number of major centres have built large cohorts of patients and have described the clinical features affecting these individuals. To a large extent, these endeavours have reinforced what was already known about the major characteristics of mitochondrial disease in adults, but a number of features had been under‐emphasised in the past. In part, this reflects the phenotypic overlap between mitochondrial disorders and common symptoms, which initially obscured the clear relationship with mitochondrial dysfunction.

Gastrointestinal (GI) features have emerged as a major element of many mitochondrial diseases (Kornblum *et al*, 2001). Both dysphagia and recurrent vomiting are now well‐recognised signs and can help to distinguish different molecular groups. For example, patients with chronic progressive external ophthalmoplegia and early dysphagia are more likely to have *RR2BM* mutations rather than a large single deletion of mtDNA as a cause of their mitochondrial disease (Pitceathly *et al*, 2012). Lower GI dysmotility is also a more common feature than was previously anticipated, with many patients describing constipation that can be very difficult to treat medically. This can lead to gastrointestinal pseudo‐obstruction and require surgical resection on occasions. Originally thought to be restricted to patients with *m*itochondrial *n*euro*g*astro*i*ntestinal *e*ncephalopathy (MNGIE) caused by *TP* mutations (Nishino *et al*, 1999), gastrointestinal signs are now well recognised to be a frequent finding in patients with m.3243A>G (Kaufmann *et al*, 2009; Nesbitt *et al*, 2013), which, like MNGIE, may present with an acute abdomen (Garcia‐Velasco *et al*, 2003; Dindyal *et al*, 2014). Despite being called the “MELAS” mutation, m.3243A>G rarely causes *m*itochondrial *e*ncephalomyopathy with *l*actic *a*cidosis and *s*troke‐like episodes (< 20% of affected mutation carriers), and much more commonly causes maternally inherited diabetes and deafness (Gorman *et al*, 2015c).

Cardiac anomalies were also under recognised in the past, and several studies have shown subclinical structural and functional abnormalities of the heart in patients with common mtDNA mutations. This has been observed in LHON (Nikoskelainen *et al*, 1994; Nemes *et al*, 2008), and m.3243A>G mutation carriers (Lodi *et al*, 2004; Vydt *et al*, 2007; Nemes *et al*, 2009; Bates *et al*, 2013). Whilst there is no doubt that some patients with mitochondrial disease develop heart failure which can be fatal if not treated effectively, the natural history of the subclinical cardiomyopathy is not well described, and it remains to be determined whether this is a major contributor to morbidity and mortality. There have also been an increased number of reports of sudden unexplained death in patients with mitochondrial disorders (Ng *et al*, 2015). Patients with Kearns‐Sayre syndrome are particularly vulnerable to heart block (Kabunga *et al*, 2015), which can cause syncope and sudden death without cardiac pacing, so it is now routine practice to monitor cardiac function in patients with mitochondrial disease with regular electrocardiography and echocardiography.

### New genotypes

The number of different genetic abnormalities found in patients with mitochondrial disease has expanded exponentially in the last decade. The widespread availability of complete mtDNA sequencing has revealed rare, but recurrent, mtDNA mutations across the globe, and a greater number of families are now known to have extremely rare or private mtDNA disorders. In one recent epidemiological study, approximately one‐third of the mtDNA mutations detected were only found in single cases (Gorman *et al*, 2015c). Although many of these patients have classical features of mitochondrial disease, some have unique or unusual phenotypes, and in some, the phenotype is organ specific.

With the exception of LHON (Wallace *et al*, 1988), and maternally inherited deafness due to the m.1555A>G mtDNA mutation (Taylor *et al*, 2003), pathogenic homoplasmic mutations were thought to be a rare cause of mitochondrial disease and not to cause multi‐system disorders. However, it is now clear that this is not the case, with several well‐documented cases of multi‐system mtDNA disease due to homoplasmic mutations (Tiranti *et al*, 2000; McFarland *et al*, 2002; Limongelli *et al*, 2004). This has been an important finding, because the presence of heteroplasmy was used as a discriminating feature in diagnostic algorithms when investigating patients with multi‐system mitochondrial disorders, and homoplasmic mutations may have been inappropriately rejected as benign polymorphisms in the past. Finally, it is now clear that mtDNA mutations are much more common in the general population than was previously thought, and are found in between 0.5 and 1% of the population at > 1% heteroplasmy levels (Elliott *et al*, 2008). In some instances, the carriers harbour low levels of mtDNA heteroplasmy, but this is not always the case, pointing towards extraneous factors—perhaps in the nuclear genome or perhaps in the environment—that contribute to the pathogenesis of mtDNA disorders in the population.

With the advent of exome and whole‐genome sequencing, novel nuclear mitochondrial disease genes have been identified at an unanticipated rate, with one or two new genes being described every month over the last 2 years. Many of these disorders present in adult life. Again, although some patients have classical mitochondrial clinical syndromes, many have discriminating clinical features or a totally distinct phenotype. Perhaps the best example is a group of patients with biochemical defects affecting multiple components of the mitochondrial respiratory chain due to a disorder of intra‐mitochondrial protein translation (Rotig, 2010; Kemp *et al*, 2011; Hallberg & Larsson, 2014; Taylor *et al*, 2014). Although some of these patients present in early childhood, this is not always the case, and a detailed consideration illustrates the emerging principles. From first principles, one would expect these patients to have a very similar phenotype because the underlying mechanism and final common pathways are identical. However, patients with *EARS2* mutations demonstrate new unique features on MRI imaging that discriminate them from other tRNA synthase disorders (Steenweg *et al*, 2012). Cardiomyopathy is particularly common in patients with *AARS2* and *MTO1* mutations (Gotz *et al*, 2011; Baruffini *et al*, 2013), which also cause a defect of intra‐mitochondrial protein synthesis. Mutations in *RMND1* cause deafness (Janer *et al*, 2012), and mutations in *YARS2* and *PUS1* cause sideroblastic anaemia (Bykhovskaya *et al*, 2004; Riley *et al*, 2010), which is unusual in mitochondrial disease. It is not clear why these differences exist, given that all of these gene defects affect intra‐mitochondrial protein synthesis. However, the dual function of some of these enzymes may provide an explanation, with phenotypic modulation mediated through cytoplasmic effects.

### Old genes new diseases

A further emerging field stems from the identification of mutations in well‐known nuclear disease genes in patients who also have a mitochondrial disorder based on conventional diagnostic criteria. *OPA1* provides one example. For over a decade, mutations in *OPA1* were known only to cause autosomal dominant optic atrophy, a very specific disorder that only causes blindness through the death of a specific cell type: the retinal ganglion cell (Alexander *et al*, 2000). However, parallel studies in Italy and the United Kingdom identified *OPA1* mutations in several families with a multi‐system mitochondrial disease (Amati‐Bonneau *et al*, 2008; Hudson *et al*, 2008). These patients not only had optic atrophy, but also developed deafness, ptosis, ophthalmoplegia, ataxia and a peripheral neuropathy in later life (Yu‐Wai‐Man *et al*, 2010b). Routine mitochondrial investigations identified multiple deletions of mtDNA in the skeletal muscle biopsies, explaining the observed mosaic cytochrome *c* oxidase defect (Yu‐Wai‐Man *et al*, 2010c). Subsequent studies in animal models strongly suggest that different components of the phenotype are caused by different mechanisms. Disrupted fusion and fission of mitochondria, linked to apoptosis, probably explains the optic neuropathy (Alavi *et al*, 2009). On the other hand, the chronic progressive external ophthalmoplegia is most likely to be due to the secondary accumulation of multiple deletions of mtDNA (Yu‐Wai‐Man *et al*, 2010b). The secondary mtDNA defects are probably an indirect consequence of the fission–fission abnormality, adding *OPA1* to the growing list of genes known to cause defects of mtDNA maintenance (discussed further below). Mutations in *OPA1* have also recently been described in families with autosomal dominant Parkinsonism (Carelli *et al*, 2015), reminiscent of the Parkinsonism described in Scandinavian families with autosomal dominant *POLG* mutations (Luoma *et al*, 2004). It is clearly important to dissect out these mechanisms if we are to develop treatments effective that tackle the problems considered to be most important by patients.

A second example is *SPG7*, known for over a decade to cause autosomal recessive hereditary spastic paraplegia (Casari *et al*, 1998). Whole‐exome sequencing in a cohort of patients with unexplained ptosis and external ophthalmoplegia, and multiple deletions of mtDNA in skeletal muscle, led to the identification of *SPG7* as a further disorder of mtDNA maintenance (Pfeffer *et al*, 2014). Intriguingly, *SPG7* appears to be the most common nuclear genetic cause of mitochondrial disease in adults to date (Gorman *et al*, 2015c; Pfeffer *et al*, 2015).

### What phenotypes are important for patients?

Although the expanding genotypic and phenotypic spectrum reinforces the long held view that mitochondrial disorders cause a complex overlapping myriad of disorders, a central question still remains: which elements are the most important for patients? As will be discussed later, this is critical for the development of new treatments for mitochondrial disease. The establishment of large cohorts of patients in Europe (http://mitocohort.ncl.ac.uk/; http://mitonet.org/) and North America (http://www.rarediseasesnetwork.org/namdc/index.htm) has provided the impetus for studies to tackle this problem. Perhaps surprisingly, muscle fatigue and weakness are at the top of the list, having the greatest impact on quality of life (Gorman *et al*, 2015a). These symptoms rarely form the focus of attention in a busy clinic (nor do they fascinate most clinicians and scientists), but ignoring the mechanisms behind these symptoms will distort priorities when it comes to the development of new treatments.

## Disease mechanisms

The identification of novel genetic causes for mitochondrial disorders has opened a Pandora's box of novel mechanisms. Initial success identifying mutations affecting the structural subunits of the mitochondrial respiratory chain (Koopman *et al*, 2012) was accompanied by the molecular dissection of the assembly appara‐tus for complexes IV (Ghezzi & Zeviani, 2012), then I (Dieteren *et al*, 2012), III (Fernandez‐Vizarra & Zeviani, 2015) and finally V (Fujikawa *et al*, 2015). Several assembly proteins also appear to have dual functions and multiple functions and remarkably, multiple functions have also been observed for the structural peptide components of complex I (Elguindy & Nakamaru‐Ogiso, 2015). What has been perhaps the most interesting is the identification of increasingly more sophisticated disease mechanisms only indirectly connected to oxidative phosphorylation and ATP synthesis. In patients presenting in adult life, disorders affecting the maintenance of mtDNA have emerged as the single most important group.

For over 25 years, it has been known that some patients with chronic progressive external ophthalmoplegia have multiple deletions of mtDNA in skeletal muscle and that the disorder can be inherited as autosomal dominant or autosomal recessive trait. In the late 1990s, reverse genetic and candidate gene studies identified mutations in *PEO1/Twinkle* (Spelbrink *et al*, 2001), encoding the mtDNA helicase; and *POLG* (Van Goethem *et al*, 2001), the mtDNA polymerase. In other families, mutations were found in the adenine nucleotide translocase gene *SLC25A4*/*ANT1* (Kaukonen *et al*, 2000). This raised the possibility that an imbalance of cytoplasmic nucleotides could cause a secondary defect of mtDNA. This mechanism was reaffirmed by the subsequent identification of autosomal recessive mutations in *TK2* (Saada *et al*, 2001), encoding thymidine kinase; and *DGUOK* (Mandel *et al*, 2001)*,* encoding deoxyguanosine kinase, in children with mtDNA depletion. The emerging pattern at this stage was of recessive mutations causing the loss of mtDNA (mtDNA depletion) and dominant mutations causing secondary mtDNA deletions and point mutations—a conclusion supported by the recent finding of single heterozygous mutations in *TK2* and *RRM2B* causing late‐onset PEO with multiple deletions in muscle (Pitceathly *et al*, 2012), and on the other hand, recessive mutations in *PEO1* causing infantile onset spinocerebellar ataxia (Hakonen *et al*, 2008). However, several examples have recently challenged this distinction. For example, patients with MNGIE typically present in early adult life with a progressive disorder mediated by a combination of both mtDNA depletion and mtDNA mutations (Mancuso *et al*, 2002; Gardner *et al*, 2015). Likewise, autosomal recessive mutations in *POLG* mediate their effect through both mtDNA depletion and secondary mtDNA mutation, as shown at the single‐cell level (Tzoulis *et al*, 2014). The balance between the depletion and secondary mutations appears to be cell and tissue specific, adding further complexity to the situation, but providing a potential explanation for the heterogeneous phenotypes.

Secondary mtDNA damage also appears to be intimately linked to mitochondrial structure, as illustrated by the multi‐system disease seen in ~20% of patients with autosomal dominant mutations in the mitochondrial fusion gene *OPA1* (Yu‐Wai‐Man *et al*, 2010b), patients with autosomal recessive mutations in *SPG7* which encodes the AAA‐protein paraplegin (Pfeffer *et al*, 2014), and dominant mutations in *AFG3L2* (Gorman *et al*, 2015b), the paraplegin binding partner. It is not clear how the secondary mtDNA defects arise in these disorders, although this appears to be intimately linked to the defective fusion and fission of the organelles themselves. Finally, recent exome studies have identified new, unrelated disease mechanisms for mtDNA maintenance disorders, including autosomal dominant mutations in *DNA2* (Ronchi *et al*, 2013), which codes for the DNA replication ATP‐dependent helicase/nuclease DNA2; and autosomal recessive mutations in *MGME1* which cause secondary mtDNA deletions by disrupting the cleavage of single‐stranded mtDNA and the processing of DNA flap substrates (Kornblum *et al*, 2013).

When considered together, these interesting findings raise several important issues for adults with mtDNA diseases. Different elements of the clinical phenotype may be caused by different mechanisms: although some may be linked to the primary function of the mutated protein, others may be due to mtDNA depletion, or the accumulation of secondary mtDNA deletions and point mutations. This raises the possibility that therapies aimed at preventing or repairing the mtDNA damage may be applicable across several disease states. The mtDNA deletions and point mutations usually require time to accumulate, explaining clinical progression and also providing a window of therapeutic opportunity. Finally, given recent reports that similar mtDNA mutations accumulate with ageing and may contribute to the ageing process (Payne & Chinnery, 2015), these disorders may be useful models to understand and slowdown the human ageing process itself.

## Natural history and biomarkers

Until recently, our understanding of the natural history of mitochondrial disease was largely based on anecdote, but longitudinal studies of large patient cohorts are now documenting clinical progression in mitochondrial disorders caused by both mtDNA and nuclear gene mutations (e.g. Grady *et al*, 2014). Although these studies are in their early stages, emerging evidence is reaffirming the view that some adult mitochondrial disorders have a fluctuating encephalopathic course, before settling into a more progressive neurodegenerative phenotype, whereas others present very much like a multi‐system neurodegenerative disorder. These data sets are providing a clearer view of the life course of a patient with mitochondrial disease. This can be helpful from a diagnostic perspective (e.g. the emergence of symptoms in a particular order may suggest one diagnosis more over another; for example, patients with ophthalmoplegia and hearing loss could have mutations in *RRM2B* or *OPA1*, but by the time the hearing loss emerges, all *OPA1* patients have an optic neuropathy). The findings will also optimise the long‐term follow‐up of patients, allowing clinicians to look for specific complications at particular stages in their disease course. The interplay between genotype and phenotype is also more complex than expected, with evidence of phenotypic modifiers for both nuclear and mtDNA disorders, and the suggestion that clinical penetrance may even decrease with subsequent generations (reverse anticipation) (Howell & Mackey, 1998). This may be in response to improving environmental conditions, although this has yet to be substantiated.

Natural history studies are being supplemented by biomarker studies. Standard diagnostic biomarkers (creatine kinase, blood and cerebrospinal fluid lactate levels) are insensitive both diagnostically, and for measuring disease progression in adults (Jackson *et al*, 1995). Serum biomarkers such as FGF21 may be useful in diagnosing childhood mitochondrial myopathy (Suomalainen *et al*, 2011), but the levels in plasma do not change over short periods of time in adults (Koene *et al*, 2014). Non‐invasive 31P‐MRS shows great potential in patients with a demonstrable bioenergetic defect in skeletal and cardiac muscle (Hollingsworth *et al*, 2012), but at present, exercise physiology and muscle biopsy are the gold standard for measuring disease progression. Unfortunately, both techniques are logistically challenging and require considerable commitment from individual patients. Hopefully, novel metabolomic profiling methods will identify the “holy grail”—a sensitive, reliable non‐invasive biomarker linked to the known disease mechanism that can be used as a surrogate in early phase experimental medicine studies testing new treatments.

## Current treatment options

The increased diagnostic yield through advanced genomics means that the majority of patients with suspected mitochondrial disease now have a molecular diagnosis. This is critically important for prognostic and genetic counselling and disease prevention, which has recently been reviewed elsewhere (Poulton *et al*, 2010). In certain circumstances, specific treatment approaches have been shown to be beneficial. These include the use of co‐enzyme Q10 in disorders of ubiquinone biosynthesis (Quinzii *et al*, 2007), allogenic bone marrow transplantation in MNGIE (Halter *et al*, 2015) and riboflavin supplementation in adults with riboflavin transporter disorders (Foley *et al*, 2014). However, despite over 1,000 articles describing possible treatments for mitochondrial disease published over five decades, there is very little objective evidence that any of the vitamins, co‐factors and dietary supplements are of any benefit in mitochondrial disorders (Pfeffer *et al*, 2012, 2013). Although this may suggest that more rigorous studies are needed, it can also be argued that the lack of any known treatment effect is evidence that these treatments are likely to only have subtle benefits at best, at least when applied across the whole patient group. It thus remains to be seen whether specific vitamins or cocktails of vitamins are helpful for defined molecular categories. One exception is idebenone, which has recently been recommended for approval by the European Medicines Agency Committee for Medicinal Products for Human Use (CHMP) for visual failure in LHON (http://www.ema.europa.eu/docs/en_GB/document_library/Summary_of_opinion_Initial_authorisation/human/003834/WC500188673.pdf). This is based on a randomised, placebo‐controlled, double‐blind, trial (900 mg/day) which failed its primary endpoint which was defined as the best recovery in visual acuity (Klopstock *et al*, 2011). However, data generated through an open‐labelled named‐patient programme convinced the reviewing committee that the treatment should be used in LHON. In most countries, there are still several hurdles to vault before idebenone can be prescribed for LHON, including obtaining approval by the health services in the various European Union member states. The development of idebenone as a treatment for LHON has taken over a decade, highlighting how complex, expensive and time‐consuming it is to take a medicine from early clinical use into clinical practice, despite the existence of “rare” or “orphan” disease legislation. Nevertheless, the published idebenone study demonstrated that multicentre, multinational randomised controlled trials are possible in mitochondrial disease, laying the foundation for the five trials ongoing in the United States studying a range of different compounds in different mitochondrial disorders (ClinicalTrials.gov) (Table 1).

**Table 1:** Registered treatment studies for mitochondrial diseases (ClinicalTrials.gov 2015)

Study title	Phase	Design	Medicine	Primary outcome(s)	Sites
Safety, Tolerability, Efficacy, PK and PD of RP103 in Children With Inherited Mitochondrial Disease (RP103‐MITO‐001)	II/III	Open Label	RP103	Change in NPMDS	USA
EPI‐743 for Mitochondrial Respiratory Chain Diseases	II	Open Label	EPI‐743	Change in Neuromuscular examination; AEs; Change in NPMDS	USA
Safety and Efficacy Study of EPI‐743 in Children With Leigh Syndrome	II	R, PC, DB	EPI‐743	Change in NPMDS	USA
A Study Investigating the Safety, Tolerability, and Efficacy of MTP‐131 for the Treatment of Mitochondrial Myopathy	I/II	R, PC, DB	MTP‐131	AEs; Change in vital signs; Changes in clinical laboratory evaluations	USA
RTA 408 Capsules in Patients With Mitochondrial Myopathy ‐ MOTOR	II	R, PC, DB	RTA408	Change in peak workload (watts/kg)	USA Denmark
EPI‐743 for Metabolism or Mitochondrial Disorders	II	R, PC, DB cross‐over	EPI‐743	Change in NPMDS	USA
Phase 2 Study of EPI‐743 in Children With Pearson Syndrome	II	Open Label	EPI‐743	Occurrence of episodes of sepsis, metabolic crisis or hepatic failure	USA
Safety Evaluation of Gene Therapy in Leber Hereditary Optic Neuropathy (LHON) Patients	I/II	Open Label	GS010 (AAV‐ND4)	Incidence of local and general adverse events and Serious Adverse Events	France
Safety and Efficacy Study of rAAV2‐ND4 Treatment of Leber Hereditary Optic Neuropathy (LHON)	I/II	Open Label	RAAV2‐ND4	Visual acuity	China
Trial of Cyclosporine in the Acute Phase of Leber Hereditary Optic Neuropathy (CICLO‐NOHL)	II	Open Label	Cyclosporine	Visual acuity	France
Safety Study of an Adeno‐associated Virus Vector for Gene Therapy of Leber's Hereditary Optic Neuropathy (LHON) Caused by the G11778A Mutation (LHON GTT)	I	Open Label	scAAV2‐P1ND4v2	AEs & SAEs	USA
MNGIE Allogeneic Hematopoietic Stem Cell Transplant Safety Study (MASS)	I	Open Label	Hematopoietic allogeneic stem cells	Neutrophil count (cells/l)	USA

AE, adverse event; DB, double blind; NPMDS, Newcastle Paediatric Mitochondrial Disease Scale; PC, placebo controlled; PD, pharmacodynamics; PK, pharmacokinetics; R, randomised.

## Conclusions

Reflecting back over the last decade, advances in molecular diagnostics have proceeded at an extraordinary pace, providing new diagnoses for singleton cases and small families. These findings are dissecting out the complex molecular mechanisms responsible for defects of oxidative phosphorylation. Large cohorts have now been assembled internationally, with over 3,000 patients being followed prospectively (see above for the web links). This has refined our understanding of phenotypic subgroups and the relationship to genotype, providing rich natural history data sets and underpinning first interventional clinical trials. Adult mitochondrial medicine has matured. We can now explain more to our patients about their disease than ever before, for some groups there are new treatment options, and there is a clear pathway towards a more comprehensive treatment in the near future.

**Pending issues**

- *Comprehensive molecular diagnosis:* With the advent of whole‐genome sequencing, a comprehensive molecular diagnosis for all patients with mitochondrial disease is within our grasp. International efforts to share data will facilitate this effort, enabling different groups to confirm or refute putative novel pathogenic mutations as early as possible.
- *Tissue selectivity:* Ultimately all mitochondrial diseases are thought to be due to a bioenergetic defect, but different mitochondrial disorders have strikingly different phenotypes. The reasons for this are not known, but are the key to understanding pathogenesis and developing new treatments.
- *Biomarker development:* Laboratory and preclinical animal studies have identified several new approaches to treat mitochondrial diseases. The major bottleneck is evaluating the potential clinical impact of these treatments in early phase human studies. Sensitive and clinically meaningful biomarkers linked to the underlying pathophysiology will accelerate this, allowing the early identification of effective and ineffective treatments.
- *Natural history studies:* There is currently a limited understanding of the natural history of mitochondrial diseases. Detailed studies of genetically homogeneous clinical cohorts are required to identify which features change over a 6‐ to 12‐month period and could be used as clinical trial endpoints. Natural history studies should be linked to biomarker development.

## Conflict of interest

The author was Chief Investigator for the Santhera Pharmaceuticals sponsored trial Rescue of Hereditary Optic Disease Outpatient Study (NCT00747487), but has remained an independent academic investigator throughout. He currently has a collaborative research project with GlaxoSmithKline on the natural history of mitochondrial disease. He has no conflict of interest with the work described in this article.
